# Physical Therapists’ Attitudes and Beliefs Regarding Treatment for Knee Osteoarthritis

**DOI:** 10.3390/jcm14197095

**Published:** 2025-10-08

**Authors:** Rami Mal, Evangelos Pappas, Hosam Alzahrani

**Affiliations:** 1 Department of Physical Therapy, College of Applied Medical Sciences, Taif University, Taif 21994, Saudi Arabia; rami.malohs@gmail.com; 2 School of Health and Biomedical Sciences, RMIT University, Melbourne, VIC 3000, Australia

**Keywords:** knee, osteoarthritis, old, physical therapy, attitudes

## Abstract

**Background/Objective:** Osteoarthritis (OA) is a progressive joint disease. Physical therapists are essential in managing OA, improving patient outcomes, and slowing disease progression, making it vital to understand their beliefs about optimal knee OA treatment. The objective is to explore physical therapists’ beliefs and attitudes toward knee OA treatment in Saudi Arabia and their alignment with guidelines. **Methods:** This cross-sectional study includes physical therapists working in Saudi Arabia who had managed at least two knee OA patients in the past six months. The survey questionnaire included questions about attitude statements, clinical management, a case study of an elderly patient with knee OA, and measurements of the level of illness perceptions and treatment choices. **Results:** This study includes 373 physical therapists (average age: 31.25 (SD 7.17); male (52.4%)). The most commonly used interventions for knee OA were strengthening exercises (19.0%) and flexibility or range of movement exercises (14.7%). About 30.4% of therapists supervised exercises in the clinic, and 89.9% provided educational advice, often focusing on weight loss, analgesia, knee support, and the use of ice or heat. Most therapists opted for treatment programs involving four to seven sessions (45.7%), with 82.2% offering follow-up care through an open appointment after discharge. **Conclusions:** The results indicate good alignment between clinical practice guidelines and physical therapists’ attitudes toward knee OA management in Saudi Arabia, though some differences exist. Therapists frequently combined exercise with educational advice on weight loss and analgesia, monitored exercise adherence, and offered follow-up care.

## 1. Introduction

Osteoarthritis (OA) is a joint disease characterized by progressive joint degeneration [1]. The joints of patients with OA demonstrate pathological changes including articular cartilage loss and damage, subchondral bone thickening, osteophyte formation, synovium inflammation, knee ligaments and menisci degeneration, and joint capsule hypertrophy [2]. Several factors can contribute to OA, such as joint trauma, obesity, older age, and genetics [3]. Patients with OA have several symptoms, including swelling, stiffness, and pain, and these can adversely impact their ability to do daily activities and consequently decrease their quality of life [4]. Globally, OA prevalence has increased by 113.25%, from 247.51 million in 1990 to 527.81 million in 2019, with a 0.12% annual increase [5]. In the Middle East and North Africa region, the prevalence of OA was 5342.8 per 100,000 people, with the highest burden in Saudi Arabia, Kuwait, and Iran [6].

Currently, several management protocols are used to control symptoms, avoid disease progression, and postpone the need for surgical intervention [7]. Exercise is commonly recommended as an effective treatment method, as it can improve physical fitness, muscle strength, and decrease symptoms [8]. The use of exercise treatment may also have benefits at the molecular level, but this can be achieved by choosing the suitable mechanism, intensity, duration, and frequency of exercise [9]. Suitable exercises can affect the extracellular matrix degradation process, joint cell apoptosis, and inflammation, which in turn relieve symptoms of OA, and reduce pain [9]. Currently, several types of exercise are used for OA including aerobics, strength, swimming, balance, proprioceptive, and neuromuscular exercises [10]. Moreover, certain types of traditional activities, such as yoga, are also used to prevent and treat OA [11].

The role of physical therapists in the management of OA is of significant importance, as their proper intervention and continuous monitoring can improve the patient’s health status and decrease the disease progression [12,13]. Moreover, the attitudes and beliefs of physical therapists can affect the quality of management programs and are of importance in the successful management of knee OA [14]. Previous studies have investigated the attitudes and beliefs of physical therapists in the context of knee OA [15,16]. Currently, there is no study that explores this question in Saudi Arabia.

International evidence indicates that, although clinicians broadly endorse exercise as core care for knee OA, beliefs and daily practice vary across settings and health systems. A recent scoping review synthesizing 12 qualitative studies—predominantly from Canada and the United Kingdom, with single studies from Australia, the Netherlands, and Denmark—reported enduring “wear-and-tear” narratives, uncertainty about the safety and effectiveness of land-based/loaded exercise, and a tendency toward conservative or low-impact options that can dilute guideline-concordant care [17]. Primary qualitative and mixed-methods studies similarly show that physiotherapists’ approaches are shaped by biomedical framing, concern about pain flares, and contextual constraints [14,15,16], while multi-stakeholder work highlights system-level barriers (time, reimbursement, referral pathways) impeding non-pharmacological care [18,19]. Contemporary recommendations therefore emphasize individualized, adequately dosed, and progressed strengthening and aerobic exercise coupled with education and behavior-change support [12,13,20]. Consistent with this, meta-analytic evidence shows that exercise dose and type matter for pain and disability [21], and large-scale implementation (e.g., GLA:D) demonstrates real-world feasibility and cost-effectiveness of structured education plus supervised exercise [22]. Support for long-term adherence—potentially including digital tools—has also been identified as an enabler of better outcomes [23,24,25,26].

Against a backdrop of substantial global and regional disease burden, no prior study has examined physiotherapists’ attitudes and beliefs regarding knee OA management in Saudi Arabia. Establishing these data and benchmarking local practice against contemporary recommendations and implementation models is therefore warranted.

This study aims to explore physical therapists’ treatment approaches for clinical knee OA in Saudi Arabia and to contextualize reported practice against contemporary recommendations for exercise delivery and implementation models.

## 2. Methods

### 2.1. Study Design and Participants

The design of this study was cross-sectional. This study included physical therapists who are currently working in Saudi Arabia in any clinical setting and managed at least one knee OA patient at the clinic in the past 6 months. It excluded non-physiotherapist healthcare professionals, undergraduate students, and those not practicing in Saudi Arabia. This study was reported following the STROBE (Strengthening the Reporting of Observational Studies in Epidemiology) guidelines to enhance the study quality and transparency of clinical research.

The protocol of this study was approved by the local ethics committee at Taif University (No. 44-106), and it was performed in accordance with the “Helsinki Declaration of 1975 as revised in 2000”. Participation in this study was voluntary, and participants were asked to give their informed consent before filling out the survey. The survey was administered using Google Forms and was distributed using a convenience sampling approach via sending the online survey to eligible participants practicing in different hospitals and outpatient clinics across Saudi Arabia. The survey was distributed via institutional e-mail lists and routine departmental communication channels used by participating hospitals/clinics. The invitation included the study description, eligibility criteria, and an informed consent statement. Eligibility was confirmed on the first survey page; ineligible individuals were screened out.

### 2.2. Sample Size

The sample size was calculated based on the statistics provided by the Saudi Arabian Commission for Health Specialties (SCFHS), where there are approximately 12,544 physical therapists working in Saudi Arabia. We assumed a margin of error of 5% and a confidence level of 95% to estimate key proportions. Using the conservative assumption *p* = 0.50, the initial requirement was *n*_0_ = (Z^2^ × *p*(1 − *p*))/d^2^ = (1.96^2^ × 0.5 × 0.5)/0.05^2^ = 384.16. Applying the finite-population correction for the national physiotherapists (*N* = 12,544) yielded *n* = (*n*_0_ × *N*)/(*n*_0_ + *N* − 1) = (384.16 × 12,544)/(384.16 + 12,544 − 1) = 372.8; thus, a minimum of 373 respondents was required. The calculation was verified using the calculator.net website, and the estimated minimum number of eligible participants was 373.

### 2.3. Survey Instrument

The survey questionnaire included two sections: the first section included several demographic questions (age, gender, nationality, highest academic qualification, years of experience, and workplace); the second section included a list of attitude statements, clinical management questions, and a case study that describes a patient with knee OA as per previous research (Supplementary Materials) [15]. Additionally, this survey questionnaire included measurements of the level of illness perceptions that was developed by Moss-Morris [27] and treatment orientations developed by Houben and Ostelo [28,29]. The survey items have been used previously by Holden et al. [15,16], but the results presented here are original to this research, which provides unique data on physiotherapists’ beliefs and attitudes towards knee OA treatment in Saudi Arabia.

### 2.4. Statistical Analysis

We used the Statistical Package for the Social Sciences (SPSS) v.23 program from IBM (Armonk, NY, USA) for the analysis. The study employed descriptive data analysis techniques, involving the computation of participants’ demographic data frequencies and percentages, and means and standard deviation (SD).

## 3. Results

### 3.1. Characteristics of Participants

The 373 participants’ characteristics are shown in Table 1. The average age of participants was 31.25 (standard deviation (SD) = 7.17). The majority of the physical therapists were males (52.4%), Saudi (86.2%), had a bachelor’s degree in physical therapy (74.1%), worked in an outpatient setting (51.6%), and had less than 6 years of clinical experience (46%).

### 3.2. Treatment Approaches and Use of Therapeutic Exercise

**Figure 1:**
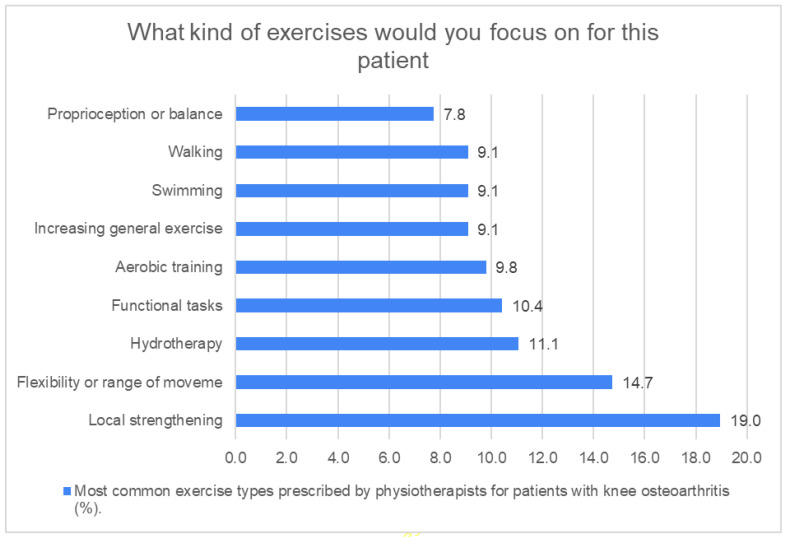
Exercises recommended by respondents’ physical therapists. Figure 1 shows the therapeutic exercise types prescribed by physical therapists. The most common types of exercise prescribed by physical therapists were local strengthening (19.0%) and flexibility or range of movement exercises (14.7%), while the least commonly used exercise was proprioception or balance (7.80%).

The treatment approaches utilized by the respondents physiotherapists for clinical knee OA are summarized in Table 2. Therapeutic exercise was the commonly utilized treatment, given that 21.0% of physical therapists have reported using it. The most frequently prescribed types of exercises by physical therapists were local muscle strengthening (19.0%), followed by flexibility or range of motion exercises (14.7%) (Figure 1). The largest number of physical therapists mentioned their supervision of exercises in the clinic (30.4%). Approximately 95.0% of respondents favored observing whether the patient was completing the desired exercise program. The highest percentage (31.3%) reported their way of checking as observation of exercise technique. The majority of respondents expressed that they would provide educational advice as part of their management (89.9%). This advice frequently concentrated on weight loss, using analgesia for pain relief, using knee support, and using ice or heat at home, with the majority advising weight loss (17.6%).

The largest percentage of physical therapists reported that they would see the patient for up to four to seven treatment sessions (45.7%). Approximately 82.2% of the respondents would provide follow-up following discharge by an open appointment. Some physical therapists expressed that they would not provide any form of follow-up due to the following reasons: the patient was anticipated to self-manage (45.3%) and/or to see another healthcare professional for follow-up (64.0%), which was predominantly an orthopedic surgeon (22.0%) or a rheumatologist (18.4%).

## 4. Discussion

This study aims to investigate physical therapists’ opinion and use of treatment options and, in particular, therapeutic exercise for knee OA management within Saudi Arabia. The findings shed light on current clinical practice and whether it matches recommendations of therapeutic exercises [13]. Aligning with those recommendations, most respondent physiotherapists reported prescribing therapeutic exercise, monitoring adherence to exercise, and offering educational advice as part of their treatment plan for knee OA patients. However, there were notable disparities between exercise guidelines and clinical practice in Saudi Arabia in terms of exercise forms, delivery methods, and adherence concerns.

In the current study, most of the physical therapists recommend therapeutic exercises, including strengthening and range of motion exercises, over other exercises, such as proprioception or balance, walking, and swimming. The existing literature shows that these exercises are effective in decreasing pain and improving function and general health in patients with knee OA [13]. Moreover, they are considered an essential approach in the knee OA patient’s therapy [20]. A previous review demonstrated that therapeutic exercise, including strengthening, functional, and stretching exercises, is effective in managing knee OA [20]. Moreover, there is evidence to recommend prescribing a variety of exercises for patients with knee OA [21]. On the other hand, in our study, approximately 92% of physiotherapists did not recommend prescribing balance exercises; however, this is in contrast to clinical guidelines, as balance exercises have been shown to positively impact muscle function and reduce disability and potentially knee OA progression [20].

Regarding exercise delivery, the majority of physical therapists in the current study reported that supervision of exercises occurs in clinics. Previous studies reported that supervised exercises are better than unsupervised exercises in improving pain and function [30]. Exercise supervision has a crucial role in improving patient safety, as it promotes proper technique, corrections, and advice. Moreover, it enables the provision of suitable dose, progression, and individual practicing and adherence of the exercise. Lack of supervision may adversely affect the overall effectiveness of the exercise program [15].

Most physical therapists planned 4–7 treatment sessions for a typical knee OA episode (45.7%), and only 14.3% planned >7 sessions. However, while the optimal number of sessions should be individualized, current implementation models recommend minimum of 12 sessions alongside patient education [22], consistent with guidance that emphasizes adequate dose and progression and supervised, active treatment as best practice [13,31]. A previous meta-analysis demonstrates that exercise type and dose affect pain and disability outcomes [21]; thus, episodes shorter than 8–12 supervised sessions may risk underdosing unless paired with a structured home-exercise plan (e.g., multiple sessions per week with explicit progression criteria) and planned follow-up reviews. In settings with capacity constraints, group classes and hybrid paths (supervised sessions plus scheduled remote sessions) can improve the progress of the exercise program dose, which in turn increases its effectiveness [13,22,31].

The exercise guidelines emphasized the significance of adherence to exercise in improving the long-term benefits of exercise in knee OA patients [13]. The majority of the respondent physical therapists in this survey, reported the observation of exercise technique as a monitoring method for exercise adherence. This method is easy and inexpensive; however, the exercise that is observed directly may not represent the exact exercise performance at home [23]. Several strategies that would improve exercise adherence in a significant way were reported. The use of an exercise diary is one of these recommended strategies that was found to have a moderate use in this study. Exercise diaries may show biases of self-report; however, they allow the self-monitoring of exercise by patients and increase patient awareness regarding exercise adherence importance in knee OA [24]. The long-term follow-up strategy was also reported by the exercise recommendations to improve exercise adherence [13,25], which had been reported by most of the physical therapists in this survey. Recently, the use of digital interventions in home exercise programs has been recommended, as it is effective in increasing exercise adherence in the short term [26]. Digital interventions can help patients in self-management and rehabilitation after injury as they promote communication with practitioners to provide the patient with needed education, advice, information, timely prompts and reminders, graded progression algorithms with goal setting, and feedback that in turn would motivate the patient toward exercise adherence. Recent syntheses and RCTs report that app-, SMS-, and platform-based adjuncts improve adherence to prescribed home exercise and can produce modest improvements in pain and function, although effects vary and adherence is not measured consistently across studies [23,24,25,26].

Although current guidelines prioritize active, supervised exercise with education as core care for knee OA, with passive modalities used as adjuncts rather than stand-alone treatments [12,13,31], about one in five (20.4%) respondents in the current study still reported utilizing electrotherapy. This pattern is probably driven by interlocking determinants including educational legacy and a persistent biomedical “wear-and-tear” schema that may heighten perceived risk of loaded, land-based exercise [17]; organizational constraints (e.g., device availability and reimbursement templates) that privilege brief device-based encounters; and patient expectations for machine-delivered analgesia. From an effective standpoint, allocating session time to passive care carries an opportunity cost, displacing teaching and progressing strengthening/aerobic work, where type and dose drive better outcomes [21]. Accordingly, de-implementation should be pursued through various strategies, including short CPD refreshers focused on individualizing and progressing load, default active-care pathways with explicit progression criteria, routine audit/feedback on the active-versus-passive modality mix, and patient-facing education to recalibrate expectations toward movement-based recovery. These steps can realign daily practice with current recommendations and improve fidelity to progression-based rehabilitation [12,13,17,21,31].

The significant role of healthcare professionals in the success of OA treatment would emphasize the importance of their understanding of current recommended treatment strategies. OA is primarily diagnosed and managed by general practitioners (GPs) who are usually unaware of the current recommended treatment strategies and would use non-evidence-based treatments such as electrotherapy in the OA treatment approach [32]. Therefore, the implementation of such recommendations in the primary-care setting would provide effective rehabilitation procedures and impede the use of low-quality and ineffective treatments [18]. Moreover, it would facilitate GPs and patient education about the current best evidence-based practices for knee OA [19].

This study has several limitations. First, recruitment utilized a convenience-sampled, online survey, which may introduce coverage and selection bias (e.g., over-representing therapists who are digitally engaged or more interested in knee OA). Therefore, estimates should be interpreted as descriptive precision targets rather than being population-weighted prevalence. Second, even though responses were anonymous and the items were neutrally phrased without any incentives, social desirability and recall/reporting bias can still influence the data because they are all self-reported in response to a clinical vignette. Third, cross-sectional design restricts interpretation of any relationships between practice choices and clinician characteristics and prohibits causal inference. Fourth, the sample’s sectoral/regional distribution and the lack of sampling weights may restrict generalizability to the national physiotherapists’ workforce. Finally, the questionnaire did not capture detailed exercise dose/progression or objective practice audits, which constrains benchmarking against delivery principles. Future research utilizing probability sampling, objective data sources (e.g., chart audit/registry), and mixed-methods designs could reduce self-report bias and strengthen external validity.

## 5. Conclusions

The current study indicated the high use of therapeutic exercise by physical therapists to treat clinical knee OA in Saudi Arabia, which is consistent with clinical guidelines. This use was also incorporated with educational advice, including weight loss and the use of analgesia. The exercise adherence was also monitored, and follow-up was offered by the majority of respondent physical therapists. On the other hand, one in five physical therapists reported that they would use electrotherapy, which is inconsistent with current evidence. Overall, the results found that there are some differences between currently used clinical practice and previously reported exercise guidelines.

## Figures and Tables

**Table 1:** Physical therapists’ demographic characteristics.

Variable	No. (%)
Age, mean (SD)	31.25 (7.17)
Sex	
Male	198 (52.4)
Female	180 (47.6)
Nationality	
Saudi	326 (86.2)
Other	52 (13.8)
Qualification	
Diploma	10 (2.6)
Bachelor	280 (74.1)
Master	70 (18.5)
Ph.D.	18 (4.8)
Clinical experience, years	
<6	174 (46.0)
6–10	105 (27.8)
>10	99 (26.2)
Work environment	
Outpatient setup	330 (51.6)
Inpatient setup	204 (31.9)
Home care	62 (9.7)
Academic institution	43 (6.8)
New patients with knee OA seen per month	
<4	147 (38.8)
4–7	153 (40.5)
>7	78 (20.6)

**Table 2:** The treatment approaches used for clinical knee OA by respondents.

Theme	No. (%)
At this point, what approaches would you use to treat this patient?	
Acupuncture	34 (2.3)
Electrotherapy	301 (20.4)
Heat or ice	293 (19.9)
Injection	23 (1.6)
Manual therapy	240 (16.3)
Rest	125 (8.5)
Tape	147 (10.0)
Therapeutic exercises	310 (21.0)
What kind of exercise would you focus on for this patient?	
Aerobic training	139 (9.8)
Flexibility or range of movement	209 (14.7)
Functional tasks	148 (10.4)
Hydrotherapy	157 (11.1)
Increasing general exercise	129 (9.1)
Local strengthening	269 (19.0)
Proprioception or balance	110 (7.8)
Walking	129 (9.1)
Swimming	129 (9.1)
How would you deliver these exercises? (either during your initial treatment session or during your follow-up session(s), if applicable)	
Refer to exercise class or group	80 (11.8)
Refer to student, assistant or technical	37 (5.4)
Supervision of exercises in clinic	207 (30.4)
Verbal advice on home exercises	196 (28.8)
Written information on home exercises	160 (23.5)
Would you check if this patient was completing the exercise program?	
Yes	288 (95.0)
No	15 (5.0)
If yes, please specify how you would do this.	
Changes in objective measures	120 (15.8)
Changes in subjective measures	91 (12.0)
Exercise diary	92 (12.1)
Observation of exercise technique	237 (31.3)
Telephone review	37 (4.9)
Verbal questioning	181 (23.9)
Would you offer any advice as part of your treatment?	
Yes	340 (89.9)
No	38 (10.1)
If yes, please specify what this advice would be	
Analgesia	230 (14.7)
Avoidance of painful movement	39 (2.5)
Increasing activity level	74 (4.7)
Nutrition	171 (10.9)
Pacing of activities	71 (4.5)
Reducing activity level	78 (5.0)
Rest	107 (6.8)
Use of ice or heat at home	203 (12.9)
Use of walking aids	113 (7.2)
Weight loss	276 (17.6)
Use of knee support	206 (13.1)
How many times would you be likely to see this patient?	
Once	15 (4.0)
2–3 times	136 (36.0)
4–7 times	173 (45.7)
>7 time	54 (14.3)
After you had discharged this patient, would you be likely to offer her any kind of physical therapy follow-up?	
Yes	249 (82.2)
No	54 (17.8)
If yes, please specify how you would do this.	
One-off follow up appointment	102 (32.5)
Open appointment	136 (43.3)
Telephone review	76 (24.2)
If no, please explain your reasons for this.	
A re-referral is needed for further treatment	13 (17.3)
I do not have this option	15 (20.0)
I expect the patient to self-manage	34 (45.3)
I would have exhausted all relevant treatment	6 (8.0)
Would you be likely to refer this patient to someone else?	
Yes	242 (64.0)
No	136 (36.0)
To whom would you refer this patient?	
Acupuncturist	6 (0.9)
Dietitian	77 (11.2)
Exercise on prescription	18 (2.6)
Family practitioner	15 (2.2)
Local pharmacist	49 (7.1)
Occupational therapist	35 (5.1)
Orthopedic surgeon	151 (22.0)
Another physical therapist	36 (5.2)
Pain clinic	111 (16.2)
Radiologist	5 (0.7)
Rheumatologist	126 (18.4)
Support group	11 (1.6)
Podiatrist	46 (6.7)

## Data Availability

The original data presented in the study are openly available in Health and Medical Care Archive at (ID: HMCA-237282).

## Supplementary Materials

The following supporting information can be downloaded at: https://www.mdpi.com/article/10.3390/jcm14197095/s1, Supplementary: Case Study—65-year-old woman with chronic left knee pain.
