# Supplementary material for: Physical Therapists’ Attitudes and Beliefs Regarding Treatment for Knee Osteoarthritis
Source: J Clin Med. 2025 Oct 8;14(19):7095. doi: 10.3390/jcm14197095 (PMC12525563; doi:10.3390/jcm14197095)
Supplement: Supplementary file 1 [file jcm-14-07095-s001.zip › jcm-3851119-supplementary.pdf]

Supplementary

Case Study: A 65-year-old woman was referred by her general practitioner with a 3-year history of left knee pain, which was of insidious onset and has gradually worsened over time. She is a retired shop manager and usually enjoys gardening, but this has become difficult due to her knee problem. Her general health is good, despite being overweight and having mild hypertension. She also has pain in both hands.

Today, she rates the intensity of her knee pain as 6 out of 10. Descending stairs, bending, and rising from sitting all aggravate her knee pain. She has some difficulty when walking and has started to use a cane outdoors. Her knee is stiff first thing in the morning and after staying in one position for too long. She finds some relief from an anti-inflammatory gel and takes up to three 200-mg ibuprofen tablets per day.

Despite not having a radiograph, she feels her problem is due to arthritis, as her father had this. It is her first referral for physical therapy, and she is optimistic about its outcome. On examination, the left knee has a mild effusion and a valgus alignment. Flexion is limited, and the quadriceps femoris muscles are weak. The joint line is tender on palpation. No other examination findings are remarkable.
